# New Insights Into the Pivotal Role of CREB-Regulated Transcription Coactivator 1 in Depression and Comorbid Obesity

**DOI:** 10.3389/fnmol.2022.810641

**Published:** 2022-02-15

**Authors:** Clara Rossetti, Antoine Cherix, Laetitia F. Guiraud, Jean-René Cardinaux

**Affiliations:** ^1^Center for Psychiatric Neuroscience, Department of Psychiatry, Lausanne University Hospital, University of Lausanne, Prilly, Switzerland; ^2^Service of Child and Adolescent Psychiatry, Department of Psychiatry, Lausanne University Hospital, University of Lausanne, Lausanne, Switzerland; ^3^Laboratory for Functional and Metabolic Imaging (LIFMET), Ecole Polytechnique Fédérale de Lausanne (EPFL), Lausanne, Switzerland

**Keywords:** major depressive disorder, obesity, circadian rhythms, neuroplasticity, CREB, CRTC1, BDNF

## Abstract

Depression and obesity are major public health concerns, and there is mounting evidence that they share etiopathophysiological mechanisms. The neurobiological pathways involved in both mood and energy balance regulation are complex, multifactorial and still incompletely understood. As a coactivator of the pleiotropic transcription factor cAMP response element-binding protein (CREB), CREB-regulated transcription coactivator 1 (CRTC1) has recently emerged as a novel regulator of neuronal plasticity and brain functions, while CRTC1 dysfunction has been associated with neurodegenerative and psychiatric diseases. This review focuses on recent evidence emphasizing the critical role of CRTC1 in the neurobiology of depression and comorbid obesity. We discuss the role of CRTC1 downregulation in mediating chronic stress-induced depressive-like behaviors, and antidepressant response in the light of the previously characterized *Crtc1* knockout mouse model of depression. The putative role of CRTC1 in the alteration of brain energy homeostasis observed in depression is also discussed. Finally, we highlight rodent and human studies supporting the critical involvement of CRTC1 in depression-associated obesity.

## Introduction

According to the World Health Organization, more than 300 million people suffer from depression worldwide (World Health Organization, 2017). This mood disorder is now the leading cause of disability and a major contributor to the overall global burden of disease. A high suicide risk is associated with severe depression and more than 800,000 people commit suicide each year. Current antidepressant therapies are mostly targeting monoamines neurotransmission with a limited efficacy. Several weeks of treatment are needed before mood improvement occurs, and approximately 30% of patients do not respond to at least two consecutive antidepressant treatments (Caraci et al., 2018). Treatment-resistant depression may benefit from the recent development of rapid-acting antidepressants targeting glutamate neurotransmission, such as the *N*-methyl-D-aspartate (NMDA) receptor blocker ketamine (Duman et al., 2016, 2019b; Zanos and Gould, 2018; Krystal et al., 2019; Shinohara et al., 2021). However, the dissociative and potentially addictive side effects of ketamine have been stimulating the development of new antidepressant drugs based on ketamine, but without its drawbacks (Ragguett et al., 2019). Unfortunately, many clinical trials failed to show a significant advantage for antidepressant medication over inert placebo (Kraus et al., 2019; Wilkinson and Sanacora, 2019). These failures might also reflect insufficient clinical subtyping of major depressive disorder (MDD), which would certainly benefit from a better understanding of the neurobiology of depression that remains elusive despite decades of intensive research (Akil et al., 2018).

Overweight and obesity are major public health problems that continue to increase both in developing and developed countries. Epidemiological evidence strongly supports the existence of a bidirectional relationship between depression and abdominal (visceral) obesity, which is the main risk factor for metabolic syndrome (Milaneschi et al., 2019). Clinically, obesity and metabolic syndrome are associated with the atypical depression subtype of MDD characterized by neurovegetative symptoms consisting of lethargy, fatigue, excessive sleepiness, increased food intake, weight gain and depressive symptoms that are lowest in the morning and worsen as the day progresses (Gold, 2015). Among the shared biological pathways that may mechanistically explain the atypical depression–obesity link, increased chronic inflammation is emerging as the central pathophysiological process involved (Milaneschi et al., 2019).

The monoamine hypothesis of depression is based on a deficit of monoamines (serotonin and noradrenaline) that would be corrected by antidepressants. However, current antidepressants require weeks of treatment to produce a clinical response, which suggests that their effects do not only rely on enhanced monoamines transmission, but rather to long-term changes in monoamines signaling and neuronal circuitry. Therefore, research efforts have been focusing on the long-term molecular changes that underlie depression and antidepressant treatments, and led to the neurotrophic and network hypotheses of depression, which proposed that impaired mechanisms of neuroplasticity are a core pathophysiological feature of MDD. Neuroplasticity is a fundamental mechanism of neuronal adaptation involving several forms of plasticity, such as adult hippocampal neurogenesis, neuronal survival and maturation, synaptogenesis, and structural plasticity (e.g., number of spines and complexity of dendritic arborization). Decreased neuroplasticity in the hippocampus and prefrontal cortex, and the resulting problems in information processing within relevant neural networks might thus underlie mood disorders (Castren, 2005; Pittenger and Duman, 2008; Marsden, 2013; Boku et al., 2018). Indeed, chronic stress strongly contributes to the development of MDD by affecting neuroplasticity and connectivity at several levels (Pittenger and Duman, 2008; Albert, 2019; Duman et al., 2019a). The transcription factor cAMP response element-binding protein (CREB) and one of its target genes, brain-derived neurotrophic factor (*BDNF*) are critically involved in the concept of altered neuroplasticity in MDD (Carlezon et al., 2005; Blendy, 2006; Castren and Hen, 2013; Levy et al., 2018; Umemori et al., 2018; Xiao et al., 2018; Castren and Monteggia, 2021). Moreover, there is evidence in rodents and humans that the CREB-BDNF pathway is also implicated in obesity (Rios, 2013; Lin et al., 2014; Marosi and Mattson, 2014; Xu and Xie, 2016; Amare et al., 2017). *BDNF* and several other neuroplasticity genes are regulated by CREB and a coactivator called CREB-Regulated Transcription Coactivator 1 (CRTC1) (Zhou et al., 2006; Kovacs et al., 2007). Recent studies suggest that CRTC1 dysregulation may be involved in the etiopathogenesis of many brain disorders, including MDD (Saura and Cardinaux, 2017). This review will focus on the increasing evidence of the key role of the transcription coactivator CRTC1 in the central control of mood and eating behavior, and its possible involvement in depression and comorbid obesity.

## CREB-Regulated Transcription Coactivator 1 in Brain Functions and Disorders

The CRTC family includes three members in mammals, with CRTC2 and CRTC3 expressed in most tissues, and CRTC1 mainly found in the brain (Kovacs et al., 2007; Li et al., 2009; Watts et al., 2011). Several comprehensive reviews already provided a detailed description of the signaling pathways regulating CRTC1 and its roles in brain physiology and pathology (Altarejos and Montminy, 2011; Escoubas et al., 2017; Saura and Cardinaux, 2017; Uchida and Shumyatsky, 2018; Parra-Damas and Saura, 2019). In this section, we summarize the mechanisms of CRTC1 regulation and its main functions in the brain, with a particular emphasis on CRTC1’s role in neuroplasticity.

**FIGURE 1:**
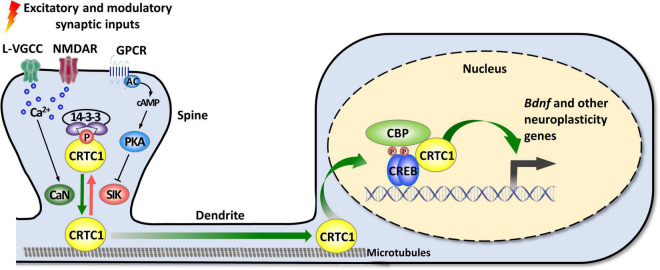
Activity-dependent synapse-to-nucleus translocation of CRTC1 mediates the activation of neuroplasticity gene transcription. CRTC1 is sequestered in dendritic spines under basal conditions via a phosphorylation-dependent association with 14-3-3 proteins. Simultaneous activation of calcium and cAMP pathways (by L-VGCC or NMDAR, and GPCR-activated AC) triggers release from 14-3-3 proteins by, respectively, activating the phosphatase calcineurin (CaN) and inhibiting kinases of the AMPK family (SIK). Dephosphorylated CRTC1 migrates into the nucleus and is recruited to the promoter via an interaction with the bZIP domain of CREB, thus promoting expression of *Bdnf* and other neuroplasticity genes. AC, adenylate cyclase; AMPK, AMP-activated protein kinase; CaN, calcineurin; CBP, CREB-binding protein; CREB, cAMP response-element binding protein; CRTC1, CREB-regulated transcription coactivator 1; GPCR, G protein-coupled receptor; L-VGCC, L-type voltage-gated calcium channels; NMDAR, *N*-methyl-D-aspartate receptor; PKA, protein kinase A; SIK, salt-inducible kinase.

As mentioned in the introduction, CRTC1 is a coactivator of CREB, a transcription factor playing pleiotropic roles in the nervous system (Carlezon et al., 2005; Barco and Marie, 2011). The canonical mode of CREB activation involves its phosphorylation at Ser133 by multiple signaling pathways, and the ensuing recruitment of CBP/p300 coactivators that activate transcription by acetylating nucleosomal histones and by interacting with factors of the general transcription machinery (Mayr and Montminy, 2001; Lonze and Ginty, 2002; Carlezon et al., 2005; Josselyn and Nguyen, 2005; Benito and Barco, 2010). However, this model was challenged by several studies suggesting that CREB can be activated without Ser133 phosphorylation (Brindle et al., 1995; Impey et al., 1996; Briand et al., 2015). The discovery of the CRTC family of coactivators brought elements of explanation about how CREB-mediated transcription could be enhanced independently of Ser133 phosphorylation, and suggested the existence of a non-canonical, alternative way of CREB target genes activation (Conkright et al., 2003; Iourgenko et al., 2003; Altarejos and Montminy, 2011; Parra-Damas et al., 2017b; Feldmann et al., 2019). *De facto*, the N-terminal domain of CRTCs interacts with the dimerization and DNA-binding bZIP domain of CREB in a phosphorylation-independent manner. Once recruited to gene promoters, CRTCs strongly activate CREB-mediated transcription through their C-terminal transactivation domain. CREB is nonetheless not constitutively activated by CRTCs, as their phosphorylation state regulates their cytoplasmic versus nuclear localization (Bittinger et al., 2004; Screaton et al., 2004; Sonntag et al., 2017). In resting conditions, they are phosphorylated and sequestered in the cytoplasm by scaffolding 14-3-3 proteins and their nuclear translocation requires the concomitant activation of calcium and cAMP signaling pathways (Figure 1). Upon elevated intracellular calcium levels, CRTCs are dephosphorylated by the Ca^2+^-dependent protein phosphatase 2B (PP2B)/calcineurin leading to their dissociation from 14–3–3 proteins and subsequent nuclear translocation. Coincidently, increased cAMP levels stimulate calcium-mediated dephosphorylation by inhibiting salt-inducible kinases (SIKs) that phosphorylate CRTCs (Altarejos and Montminy, 2011).

In neurons, synaptic activity-induced CRTC1 translocation from synapses to the nucleus is critical for the transcription-dependent phase of neuronal plasticity (Zhou et al., 2006; Kovacs et al., 2007; Li et al., 2009; Finsterwald et al., 2010; Ch’ng et al., 2012, 2015; Nonaka et al., 2014; Parra-Damas et al., 2014, 2017a). Activity-dependent transport of CRTC1 from dendritic spines to the nucleus requires local elevation of calcium triggered by activation of glutamate receptors and L-type voltage-gated calcium channels, leading to calcineurin activation and dephosphorylation of three conserved serine residues (S64, S151, and S245) in the amino-terminal third of CRTC1, which contains a nuclear localization signal (Ch’ng et al., 2015). Dephosphorylated CRTC1 is released from 14-3-3 proteins and actively translocated to the nucleus via a dynein motor protein-mediated retrograde transport along microtubules. Nuclear CRTC1 upregulates the expression of genes that have been involved in synaptic plasticity and memory formation. These neuroplasticity genes include, among others, the neurotrophic factor *Bdnf*, the immediate-early genes *c-fos*, *Zif268/Egr1*, and *Arc*, the orphan nuclear receptors *Nr4a1* and *Nr4a2*, the brain-specific growth factor *Fgf1*, and autophagy genes involved in synaptic turnover and late-phase long-term synaptic depression (Zhou et al., 2006; Espana et al., 2010; Breuillaud et al., 2012; Ch’ng et al., 2012, 2015; Nonaka et al., 2014; Parra-Damas et al., 2014, 2017a; Fukuchi et al., 2015; Uchida et al., 2017; Pan et al., 2021). In agreement with CRTC1’s role in the induction of these neuroplasticity genes, accumulating evidence supports its involvement in neuronal plasticity processes, such as activity- and BDNF-induced dendritic growth of developing cortical neurons (Li et al., 2009; Finsterwald et al., 2010), maintenance of hippocampal late-phase LTP (Zhou et al., 2006; Kovacs et al., 2007; Uchida et al., 2017), and long-term memory formation (Sekeres et al., 2012; Nonaka et al., 2014; Parra-Damas et al., 2014, 2017a; Uchida et al., 2017; Zhang et al., 2019; Shu et al., 2020; Yan et al., 2021). Of particular importance, increasing evidence suggests that CRTC1 dysregulation may be implicated in the etiopathogenesis of neurodegenerative diseases, such as Huntington’s, Parkinson’s, and Alzheimer’s disease, as well as psychiatric disorders (Boulting et al., 2021), including mood disorders [reviewed in Saura and Cardinaux (2017)].

## Emerging Role of CREB-Regulated Transcription Coactivator 1 in the Pathophysiology of Major Depressive Disorder

In line with CRTC1’s role in neuroplasticity processes, as well as the involvement of CREB and BDNF in rodent models of depression, increasing evidence suggests that CRTC1 is implicated in the pathogenesis of MDD (summarized in Table 1). Originally, we developed a CRTC1 knockout mouse model (Breuillaud et al., 2009), and performed its in-depth phenotypic characterization (Breuillaud et al., 2012). This led us to provide the first preclinical evidence of the involvement of CRTC1 in mood regulation and antidepressant response. Indeed, *Crtc1^–/–^* mice exhibit depressive-like neurobehavioral endophenotypes, including increased behavioral despair in the forced swim test (FST) and in the repeated open-space forced swim test (OSFST), social withdrawal, decreased sexual motivation, psychomotor retardation, and increased emotional response to stressful events (Breuillaud et al., 2012). High-performance liquid chromatography (HPLC) monitoring of the levels of monoamines and metabolites in the prefrontal cortex, hippocampus, hypothalamus, and nucleus accumbens revealed significantly lower levels of the dopamine metabolites 3,4 dihydroxy-phenylacetic acid (DOPAC) and homovanillic acid (HVA), as well as of the serotonin metabolite 5-hydroxyindole acetic acid (5-HIAA) in the prefrontal cortex of *Crtc1^–/–^* male mice as compared to wild-type littermates. Decreased levels of these metabolites are thought to reflect a reduced dopamine and serotonin release in the prefrontal cortex that was previously associated with aggressive behaviors, impulsivity, and depressive-like behaviors. Moreover, *Crtc1^–/–^* male mice have a decreased expression of several neuroplasticity genes in the prefrontal cortex and hippocampus, including *Bdnf* and its receptor *TrkB*, as well as the nuclear receptors *Nr4a1-3*, thus suggesting impaired neuroplasticity processes in these brain structures. Interestingly, we showed that *Crtc1^–/–^* mice are resistant to the chronic antidepressant effects of the selective serotonin reuptake inhibitor fluoxetine, as well as the tricyclic antidepressant desipramine, in a behavioral despair paradigm (OSFST), which suggests that CRTC1 is required for conventional antidepressant therapy (Breuillaud et al., 2012; Meylan et al., 2016b). Supporting the blunted behavioral response to chronic desipramine, this antidepressant does not increase *Bdnf* expression in the prefrontal cortex of *Crtc1^–/–^* mice in contrast to its upregulation in wild-type mice. Moreover, we showed that chronic systemic administration of the histone deacetylase (HDAC) inhibitor suberoylanilide hydroxamic acid (SAHA, also known as vorinostat) partially rescues the depressive-like behavior of *Crtc1^–/–^* mice and restores *Bdnf* expression in the prefrontal cortex (Meylan et al., 2016b). The rationale behind this epigenetic intervention was that CRTC1 helps phosphorylated CREB to recruit the histone acetyltransferase CBP and that the absence of CRTC1 decreases CBP recruitment and histone acetylation of neuroplasticity gene promoters involved in mood regulation. Reduction of CBP recruitment and histone acetylation in *Crtc1^–/–^* mice can thus be partially overcome by HDAC inhibition, which restores the expression of a subset of genes by acting downstream of CRTC1. These findings are in line with the current understanding of the involvement of epigenetic processes in the pathogenesis of MDD and antidepressant action, as well as with the recent evidence of the therapeutic potential of HDAC inhibitors (Vialou et al., 2013; Misztak et al., 2018; Uchida et al., 2018).

**TABLE 1:** Summary of studies implicating CRTC1 in the pathophysiology of major depressive disorder (MDD).

References	System	Findings
Breuillaud et al., 2012	*Crtc1^–/–^* mice	Depressive-like behaviors in *Crtc1^–/–^* males: increased behavioral despair (FST, OSFST), decreased sexual motivation, anhedonia, social withdrawal. Blunted antidepressant response to the selective serotonin reuptake inhibitor fluoxetine. Decreased dopamine and serotonin turnover in the prefrontal cortex (PFC). Decreased expression of neuroplasticity genes, including *Bdnf*, its receptor *TrkB*, the nuclear receptors *Nr4a1-3*, and several other CREB-regulated genes in the hippocampus (HIP) and PFC.
Meylan et al., 2016a	*Crtc1^–/–^* mice	*Crtc1^–/–^* male mice not responding to the tricyclic antidepressant desipramine in a behavioral despair paradigm (OSFST). *Crtc1^–/–^* male mice responding to the antidepressant effects of chronic SAHA administration (HDAC inhibitor). Epigenetic rescue of *Bdnf* expression in the PFC.
Meylan et al., 2016b	*Crtc1^–/–^* mice	Upregulation of the agmatine-degrading enzyme agmatinase in the HIP and PFC of *Crtc1^–/–^* male and female mice. Acute agmatine and ketamine treatments comparably improved the behavioral despair (FST) of *Crtc1^–/–^* male and female mice. NMDA receptor antagonist properties of agmatine possibly underlying its fast-acting antidepressant effect.
Jiang B. et al., 2019	CSDS and CUMS mouse models of depression	Increased expression of hippocampal SIK2 by chronic stress (CSDS and CUMS), leading to reduced CRTC1 nuclear translocation and *Bdnf* expression, as well as decreased levels of hippocampal CRTC1. AAV-mediated overexpression of SIK2 in the HIP of non-stressed mice induced depressive-like behaviors in the FST, tail suspension test, sucrose preference test, and social interaction test. Hippocampal SIK2 overexpression reduced nuclear and total CRTC1 levels, downregulated BDNF and its signaling cascade, and decreased adult hippocampal neurogenesis. AAV-shRNA-mediated knockdown of hippocampal SIK2 or genetic knockout of *Sik2* protected mice in CSDS and CUMS models of depression with antidepressant-like effects depending on the CRTC1-CREB-BDNF pathway. Fluoxetine, venlafaxine and mirtazapine, belonging to three different antidepressant classes, all reversed the effects of CUMS and CSDS on hippocampal SIK2 and CRTC1, and their antidepressant actions were fully abolished by hippocampal CRTC1 silencing.
Ni et al., 2019	LPS-induced mouse model of depression	AAV-shRNA-mediated downregulation of CRTC1 induced depressive-like behaviors in naive mice and a decreased expression of BDNF and VGF in the ventral HIP. AAV-mediated CRTC1 overexpression in the ventral HIP prevented LPS-induced depressive-like behaviors, and restored BDNF and VGF levels. Upregulation of CRTC1 in the ventral HIP decreased LPS-induced pro-inflammatory cytokines (IL-6, IL1β, TNFα).
Liu et al., 2020	CSDS and CUMS mouse models of depression	ARN-3236, a selective inhibitor of SIK2, induced significant antidepressant-like effects in both the CSDS and CUMS models of depression by acting on the hippocampal CRTC1-CREB-BDNF pathway and adult hippocampal neurogenesis.
Wang et al., 2020	3 chronic stress mouse models of depression	Imipramine, a tricyclic antidepressant, reversed the down-regulating effects of chronic restraint stress (CRS), CUMS and CSDS on CRTC1 expression in the medial PFC but not in the HIP. AAV-shRNA-mediated downregulation of CRTC1 in the medial PFC fully abolished the antidepressant-like actions of imipramine in the FST, tail suspension test, and sucrose preference test.
Cherix et al., 2020	*Crtc1^–/–^* mice	*In vivo* neuroimaging techniques, such as high field magnetic resonance imaging (MRI), spectroscopy (MRS) and positron emission tomography (PET) revealed an altered glucose metabolism and low energetic status in the HIP of *Crtc1^–/–^* male mice resulting in excessive GABAergic neurotransmitter cycling and depressive-like behaviors. Restoration of hippocampal energy balance with ebselen, an energy-boosting mood-stabilizer, rescued the behavioral despair (OSFST) of *Crtc1^–/–^* male mice.
Si et al., 2021	Prenatally stressed male offspring rats	Depressive-like behaviors (FST and sucrose preference test) of adult male offspring rats from mothers exposed to CRS during pregnancy were associated with decreased levels of total CRTC1, nuclear CRTC1, calcineurin, BDNF and c-fos in the HIP and PFC. Chronic fluoxetine treatment (from postnatal days 30–51) reversed both the depressive-like behaviors and the downregulation of the CRTC1-BDNF pathway of prenatally stressed rats.

Using a cDNA microarray approach, we identified differentially expressed genes in the cortex of *Crtc1^–/–^* mice, among which we found an upregulation of agmatinase, the enzyme degrading the arginine-decarboxylation product agmatine (Meylan et al., 2016a). Increasing evidence suggests that agmatine is an important neuromodulator, possibly even serving as a neurotransmitter, as it is stored in synaptic vesicles and released upon depolarization, selectively recaptured and finally degraded by agmatinase to form putrescine, a key precursor for polyamine synthesis (Li et al., 1994; Reis and Regunathan, 2000; Uzbay, 2012; Piletz et al., 2013). Agmatine is primarily found in neurons of brain regions that subserve cognition, processing of emotions, visceral and neuroendocrine control, and pain perception, as the distribution of agmatine-containing neurons is concentrated in the cerebral cortex, hippocampus, amygdala, septum, bed nucleus of the stria terminalis, periventricular thalamic and hypothalamic nuclei, as well as several areas of the midbrain and brainstem (Reis and Regunathan, 2000). Agmatine has been colocalized with other neurotransmitters, notably glutamate in synaptic terminals in hippocampal CA1 region (Seo et al., 2011). It also binds with high affinity several postsynaptic membrane receptors, such as α2-adrenergic receptors, imidazoline I1 and I2 receptors, nicotinic receptors and serotoninergic 5HT-2A and 5HT-3 receptors, and blocks glutamate NMDA receptor channels in a similar way as the rapid-acting antidepressant ketamine (Yang and Reis, 1999; Reis and Regunathan, 2000; Halaris and Piletz, 2007; Uzbay, 2012; Neis et al., 2016a,b, 2018; Barua et al., 2019; Camargo and Rodrigues, 2019; Valverde et al., 2021). Moreover, agmatine is a key modulator of nitric oxide and polyamine overproduction during the “Polyamine Stress Response” that is important for proper adaptive responses of quiescent cells to stressful conditions (Piletz et al., 2013). Accumulating evidence implicates the agmatinergic system in the etiopathogenesis of mood disorders (Laube and Bernstein, 2017; Weiss et al., 2018; Watts et al., 2019) and other central nervous system disorders (Neis et al., 2017). Post-mortem studies showed a strong upregulation of agmatinase levels in hippocampal GABAergic interneurons of brain tissues from depressed subjects (Bernstein et al., 2012), and decreased agmatine levels in the cortex of individuals who died by suicide (Chen et al., 2018). Acute and chronic stress reduce agmatine levels in rodent forebrain and exogenous agmatine administration has rapid antidepressant effect in animal models of depression (Zomkowski et al., 2002; Freitas et al., 2016). Consistent with their common property to block glutamate NMDA receptors, we found that acute agmatine and ketamine treatments comparably improve the depressive-like phenotype of male and female *Crtc1^–/–^* mice in the forced swim test (Meylan et al., 2016a). Current understanding of the mechanisms underlying ketamine’s rapid antidepressant action involves direct and indirect downstream consequences of NMDA glutamate receptor antagonism (Duman et al., 2019b; Krystal et al., 2019). The indirect hypothesis of the antidepressant actions of ketamine implies that ketamine initially blocks NMDA receptors on GABA inhibitory interneurons leading to “indirect” disinhibition of glutamate transmission and enhanced stimulation of AMPA glutamate receptors on excitatory neurons. AMPA receptor activation triggers a signaling cascade that raises BDNF levels. TrkB receptors activated by the local release of BDNF stimulate the activation of the molecular target of rapamycin complex 1 (mTORC1), which, in turn, catalyzes local protein synthesis necessary for increasing dendritic spine formation and restoring synaptic connectivity. The second hypothesis of ketamine’s action is based on the direct inhibition of NMDA receptors located on excitatory neurons, and the involvement of the eukaryotic elongation factor 2 (eEF2) kinase pathway. Ketamine blockade of NMDA receptors at rest leads to suppression of eEF2 kinase activity and subsequent decreased eEF2 phosphorylation that activates molecular target of rapamycin (mTOR)-regulated local protein synthesis. The resulting increased synthesis of BDNF and other postsynaptic proteins stimulates the shuttling of AMPA glutamate receptors to the synapse, which enhances synaptic efficacy (Autry et al., 2011). In our study, we found that agmatine rapidly increases BDNF levels only in the prefrontal cortex of wild-type females, and decreases eEF2 phosphorylation in the prefrontal cortex of male and female wild-type mice, indicating that agmatine might function as a fast-acting antidepressant with NMDA receptor antagonist properties. In contrast to wild-type mice, the acute antidepressant effects of agmatine do not seem to rely on eEF2 dephosphorylation-dependent increase of BDNF synthesis in *Crtc1^–/–^* mice. These findings support a causal role of the deregulated agmatinergic system in the *Crtc1^–/–^* mouse model of depression, as it has been suggested for human MDD. On the other hand, the mechanistic relationship between agmatinase upregulation and *Crtc1* deficiency is not clear yet and deserves more investigation.

Recently, several studies highlighted the importance of CRTC1 in stress- and inflammation-induced depressive-like behaviors, thus extending our pioneering findings on the role of CRTC1 in MDD (Davis, 2019; Jiang B. et al., 2019; Ni et al., 2019; Liu et al., 2020; Wang et al., 2020; Si et al., 2021). In a quite extensive study, the SIK2-CRTC1-CREB-BDNF pathway was proved highly instrumental in mediating chronic stress-induced depressive-like behaviors and antidepressant response in C57BL/6J mice (Jiang B. et al., 2019). Both chronic unpredictable mild stress (CUMS) and chronic social defeat stress (CSDS) markedly increase hippocampal *Sik2* expression, which reduces CRTC1 nuclear translocation and *Bdnf* expression, as well as total protein levels of hippocampal CRTC1. Adeno-associated virus (AAV)-mediated overexpression of SIK2 in the hippocampus of non-stressed mice induces depressive-like behaviors in the FST, tail suspension test, sucrose preference test, and social interaction test, thus mimicking the effects of chronic stress. Hippocampal SIK2 overexpression lowers nuclear and total CRTC1 levels, downregulates BDNF and its signaling cascade, and decreases adult hippocampal neurogenesis. Conversely, AAV-short hairpin RNA (shRNA)-mediated knockdown of hippocampal SIK2 or genetic knockout of *Sik2* protect mice against both chronic stress models of depression with antidepressant-like effects that require the downstream CRTC1-CREB-BDNF pathway. Finally, fluoxetine, venlafaxine and mirtazapine, belonging to three different antidepressant classes, all reverse the effects of CUMS and CSDS on hippocampal SIK2 and CRTC1, and their antidepressant actions are fully abolished by hippocampal CRTC1 silencing. Two follow-up studies from the same laboratory then showed that the antidepressant effects of imipramine depend on CRTC1 in the medial prefrontal cortex (Wang et al., 2020), and that ARN-3236, a selective inhibitor of SIK2, induced significant antidepressant-like effects in both the CSDS and CUMS models of depression by acting on the hippocampal CRTC1-CREB-BDNF pathway (Liu et al., 2020). Moreover, it was recently shown that prenatally stressed offspring male rats display depressive-like behaviors associated with decreased levels of CRTC1 and BDNF in the hippocampus and prefrontal cortex, which are restored by a chronic fluoxetine treatment (Si et al., 2021). Taken together, these findings strengthen the pivotal role of hippocampal and prefrontal CRTC1 in the pathogenesis of MDD and in antidepressant response.

Another study convincingly highlighted CRTC1’s key role in lipopolysaccharide (LPS)-induced depressive-like behaviors (Ni et al., 2019). Systemic administration of LPS causes chronic neuroinflammation and depressive-like behaviors in mice (Dantzer et al., 2008). This animal model is relevant for the human neuroinflammation hypothesis of MDD that is associated with the atypical depression subtype (Woelfer et al., 2019). Starting from the observation that a concentration of LPS inducing depressive-like behaviors in ICR mice also decreases hippocampal CRTC1 protein levels, Ni et al. (2019) used AAV-mediated silencing or upregulation of CRTC1 in the ventral hippocampus to determine its role in LPS-induced depressive-like behaviors. AAV-shRNA-mediated downregulation of CRTC1 induces depressive-like behaviors in naive mice and a decreased expression of BDNF and VGF in the ventral hippocampus. Conversely, ventral hippocampal AAV-mediated CRTC1 overexpression prevents depressive-like behaviors induced by a single i.p. injection of LPS, and restores BDNF and VGF levels. Interestingly, upregulation of CRTC1 in the ventral hippocampus interferes with neuroinflammatory processes, as shown by the dampened accumulation of LPS-induced pro-inflammatory cytokines, such as interleukin-6, interleukin 1-β and tumor necrosis factor α. These compelling findings further support the essential role of CRTC1 in MDD pathogenesis, and suggest that CRTC1 coactivates genes controlling neuroinflammation through still unknown mechanisms (Davis, 2019; Ni et al., 2019).

Lately, ketamine has attracted much attention for its rapid antidepressant effects in patients with treatment-resistant depression (Kraus et al., 2019). Ketamine and a few other compounds acting on glutamate neurotransmission are the only antidepressant drugs in development that are able to relieve MDD symptoms within hours after a single dose, the effects of which lasting for up to a week. Unfortunately, ketamine is also a well-known psychedelic drug with an addictive potential, and almost nothing is known about the risks of its long-term use. Current research is thus focusing on the development of ketamine-like molecules having the same fast-acting antidepressant benefits without the undesirable side effects (Zanos et al., 2018; Ragguett et al., 2019; Wilkinson and Sanacora, 2019). This preclinical development of novel pharmacotherapies requires a better understanding of the molecular and cellular mechanisms underlying the antidepressant actions of ketamine. A recent study showed that ketamine’s acute antidepressant effects precede its action on spine formation in the prefrontal cortex, indicating that spinogenesis is not required for the rapid (less than 12 h) behavioral response (Moda-Sava et al., 2019). In contrast, prefrontal cortical restoration of lost spines by a single ketamine injection is necessary for its sustained (2–7 days) antidepressant effects. BDNF and its receptor TrkB, as well as VGF, have been implicated in ketamine’s antidepressant effects (Autry et al., 2011; Bjorkholm and Monteggia, 2016; Ma et al., 2017; Song et al., 2017; Zanos and Gould, 2018; Jiang C. et al., 2019). However, their respective function in the acute and/or sustained effects of ketamine is not completely understood. The involvement of CRTC1 in the regulation of BDNF and VGF expression (Zhou et al., 2006; Parra-Damas et al., 2014; Fukuchi et al., 2015; Ni et al., 2019), as well as the decreased brain BDNF levels in *Crtc1^–/–^* mice (Breuillaud et al., 2012; Meylan et al., 2016a), suggest that ketamine’s antidepressant effects might rely on CRTC1, as well. We previously showed that the immobility of *Crtc1^–/–^* males and females is significantly decreased 30 min after a single injection of ketamine (3 mg/kg) in the FST paradigm [Supplementary Figure 2 in Meylan et al. (2016a)], thus indicating that CRTC1 is not required for the acute antidepressant effects of ketamine. Future investigations are nevertheless needed to determine whether CRTC1 is involved in the neuroplasticity-related sustained long-term antidepressant effects of ketamine. In summary, our findings, as well as several subsequent studies, highlight the pivotal role of the CRTC1-CREB-BDNF pathway in preclinical animal models of MDD, and provide evidence of its involvement in antidepressant response.

## Deregulation of Brain Energy Homeostasis in Major Depressive Disorder: Possible Role of CREB-Regulated Transcription Coactivator 1

Several lines of evidence suggest that altered energy metabolism underlies mood disorders, both at the brain and peripheral level (Ostergaard et al., 2018). The rationale being that normal neuronal function is challenged in MDD due to an imbalance in energy homeostasis, through either an impaired glycolytic (Videbech, 2000) or mitochondrial ATP production (Klinedinst and Regenold, 2015). This imbalance is exacerbated by excessive energy demand, typically occurring during stress exposure (Picard et al., 2018). Several magnetic resonance spectroscopy (MRS) and positron-emission tomography (PET) studies have identified key brain regions with abnormal metabolic activity. However, it appears that systemic metabolic mechanisms play a role as well, associating several endocrine dysregulations and inflammatory processes with impaired neuroenergetics. An example of this association is the relation of metabolic syndrome to atypical depression, as highlighted by their high comorbidity (Marazziti et al., 2014). Metabolic syndrome is a cluster of health conditions including abdominal obesity, high blood pressure and insulin resistance, which reflect impaired energy utilization and storage. In fact, MDD has been associated with impaired glucose homeostasis, and in particular insulin resistance [for a systematic review and meta-analysis see Kan et al. (2013)], which can occur in the brain itself (Watson et al., 2018; Lyra et al., 2019). Brain insulin resistance has thus become a therapeutic target for treating MDD and its associated symptoms (Hamer et al., 2019). For instance, insulin sensitizers have proven antidepressant efficacy in animal models (Zhao et al., 2016) and patients (Sepanjnia et al., 2012; Kemp et al., 2014) leading to improved glucose metabolism (Lin et al., 2015). Among the numerous possible causes, stress appears to be a key modulator, if not initiator, of such metabolic disturbances. Notably, insulin resistance is a recognized consequence of allostatic overload triggered by stress (Rasgon and McEwen, 2016) and has been associated with depressive phenotype (Li et al., 2013; van der Kooij et al., 2018; Yang et al., 2018).

Control of energy homeostasis is complex and involves several energy-sensing molecules. Intracellular detectors like AMPK (AMP-activated kinase), SIRT1 (Sirtuin 1) or HIF (Hypoxia-inducible factor), provide a rapid response to fluctuations in ATP, NADH and oxygen concentrations, and have been implicated in MDD (Ostergaard et al., 2018). Intercellular signaling, in a paracrine, endocrine or neurotransmission fashion (e.g., through monoamines, glucocorticoids or insulin), is another key mechanism for regulating metabolic activity based on energy demand. As such, the cAMP-CREB pathway is central to metabolic homeostasis by stimulating tissue-specific gene expression, through its extracellular metabotropic signaling sensitivity. CRTCs, as co-activators of CREB, play a central modulatory role in this cascade (Altarejos and Montminy, 2011). Hepatic CRTC2 promotes gluconeogenic response through opposite action of insulin or glucagon on SIK2. During short-term fasting, glucagon-induced rise in hepatic cAMP triggers the dephosphorylation of CRTC2, whereas upon feeding, the insulin-dependent activation of Akt leads to CRTC2 phosphorylation and cytoplasmic retention (Altarejos and Montminy, 2011). CRTC2 also holds a regulatory role on insulin secretion in the β-cells of the pancreatic islets (Screaton et al., 2004). In contrast, CRTC3 is mainly found in adipose tissue, where it enhances insulin resistance and obesity (Song et al., 2010). Finally, in skeletal muscles, the CRTC-CREB pathway seems to play a role in mitochondrial biogenesis through the expression of peroxisome proliferator activated receptor-γ (PPARγ) co-activator 1α (PGC1α) (Wu et al., 2006). CRTC1 is primarily expressed in the central nervous system and particularly abundant in limbic areas of mammalian brain. Neuronal CRTC-1 is controlling mitochondrial metabolism in *Caenorhabditis elegans* (Burkewitz et al., 2015). Likewise, the SIK-CRTC1 pathway was identified as an important cascade for controlling neuronal energy homeostasis in *Drosophila* (Choi et al., 2011; Shen et al., 2016). Studies in rodents highlight the importance of the neuronal CRTC1-CREB pathway in translating metabotropic neurotransmission signaling into synaptic plasticity (Saura and Cardinaux, 2017). While it seems clear that synapse formation and plasticity require stimulation of energy metabolism, the mechanistic pathway involving CRTC1 remains to be discovered in the mammalian brain. The role of CRTC1 in controlling energy homeostasis appears to be complex and multifactorial, involving both peripheral and central control. In fact, despite being primarily expressed in the brain, CRTC1 plays a role in peripheral tissues as well (Kim et al., 2015; Kim, 2016; Schumacher et al., 2016; Gao et al., 2018; Morhenn et al., 2019). Deletion of *Crtc1* gene in mice induces insulin resistance and obesity, together with a depressive-like phenotype (Altarejos et al., 2008; Breuillaud et al., 2009, 2012; Rossetti et al., 2017). Using *in vivo* MRS at high field, we have observed that this phenotype translates into measurable neurochemical alterations in the hippocampus of *Crtc1^–/–^* mice (Cherix et al., 2020). Noteworthy, we identified lower levels of phosphocreatine relative to creatine, an indication that ATP production or consumption is impaired. These results suggest that CRTC1 is required for normal energy homeostasis in the brain and could mechanistically underlie similar observations arising from MRS studies in human depression (Moore et al., 1997; Harper et al., 2017). Finally, the strong resemblance between the *Crtc1^–/–^* mouse phenotype and metabolic syndrome might provide new avenues for exploring how energy deregulations relate to human MDD (Rasgon and McEwen, 2016; Watson et al., 2018).

## CREB-Regulated Transcription Coactivator 1 in Major Depressive Disorder-Associated Obesity

Epidemiological evidence clearly suggests a comorbid association between depression and obesity (Amare et al., 2017; Milaneschi et al., 2019). Indeed, recent cross-sectional and longitudinal meta-analyses have demonstrated a positive and bidirectional relationship between these two pathological conditions. Moreover, these meta-analyses indicate that the MDD-obesity comorbidity occurs in both adulthood and adolescence, that it is not related to sociodemographic and lifestyle factors, and cannot be completely explained by the body weight increase induced by antidepressant medications. Compared to the general depression-obesity association, even stronger comorbidity was observed in atypical depressed patients, a subgroup of MDD patients showing sustained food intake during depressive episodes, increased visceral fat deposition and adverse metabolic profile (Xu et al., 2011). In keeping with this observation, a recent investigation suggests that the association between atypical depressive symptoms and obesity-related traits may arise from shared pathophysiological mechanisms (Milaneschi et al., 2017).

The wide distribution of CRTC1 in the rodent brain has been related to several functions spanning from synaptic plasticity, learning and memory, emotional processing and central energy-balance regulation (Saura and Cardinaux, 2017). In accordance with this broad function, the genetic suppression of CRTC1 in the mouse was found to induce depressive-like symptoms and obesity (Altarejos et al., 2008; Breuillaud et al., 2009, 2012; Rossetti et al., 2017). Although the human *CRTC1* polymorphisms studied so far were not clearly associated with depression, they were associated with obesity markers [body mass index (BMI), fat mass] in psychiatric cohorts and in individuals with MDD (Choong et al., 2013; Quteineh et al., 2016). With regard to obesity, further human genetic investigations showed that the *CRTC1* locus links fat mass to cardiometabolic diseases (Lu et al., 2016) and that genetic and epigenetic control of *CRTC1* transcription affects fat distribution and eating behavior (Booij, 2019; Delacretaz et al., 2019; Rohde et al., 2019; Wang et al., 2021). Altogether, these observations strengthen the hypothesis that CRTC1 may represent a pivotal transcription coactivator regulating both MDD and obesity etiological pathways (see Tables 1, 2).

**TABLE 2:** Summary of studies implicating CRTC1 in obesity.

References	System	Findings
Altarejos et al., 2008	*Crtc1^–/–^* mice	*Crtc1^–/–^* mice are hyperphagic, obese and infertile. Hypothalamic CRTC1 phosphorylated and inactive in leptin-deficient ob/ob mice, and leptin administration increased dephosphorylated nuclear CRTC1. CRTC1 regulates the *Cartpt* and *Kiss1* genes, which encode hypothalamic neuropeptides that mediate leptin’s effects on satiety and fertility.
Breuillaud et al., 2009	*Crtc1^–/–^* mice	*Crtc1^–/–^* mice are obese, but not infertile. No alteration of hypothalamic *Kiss1* gene expression and plasma luteinizing hormone levels.
Kim et al., 2015	Streptozotocin-induced (STZ) diabetic *Crtc1*^+/−^ mice	Leptin improved diabetic glucose metabolism through *Crtc1*-dependent and independent mechanisms. Leptin reduced diabetic hyperglycemia, hepatic gluconeogenic gene expression and selectively increased glucose disposal to brown adipose tissue and heart, in STZ-diabetic WT mice but not *Crtc1*^+^*^/–^* mice. Leptin promoted CRTC1 nuclear translocation in pro-opiomelanocortin (Pomc) and non-Pomc neurons within the hypothalamic arcuate nucleus, and leptin-induced Pomc gene expression was blunted in STZ-diabetic *Crtc1*^+^*^/–^* mice.
Rossetti et al., 2017	*Crtc1^–/–^* mice	Gender difference in the homeostatic regulation of energy balance. *Crtc1^–/–^* males are hyperphagic and rapidly develop obesity on normal chow diet. *Crtc1^–/–^* females exhibit mild late-onset obesity without hyperphagia. Alterations in the expression of several orexigenic and anorexigenic hypothalamic genes in mutant males. *Crtc1^–/–^* males’ hyperphagic behavior is restricted to the diurnal (resting) phase of the light cycle during which they have a higher locomotor activity.
Matsumura et al., 2021	*Sf1*-cre *– Crtc1*^loxP/loxP^** mice	Mice with a ventromedial hypothalamus (VMH)-specific knockdown of *Crtc1* are sensitive to high-fat diet-induced obesity, exhibiting hyperphagia and increased body weight gain. Unlike *Crtc1^–/–^* mice, VMH-specific *Crtc1* deletion did not affect body weight gain or food intake in normal chow feeding.
Hu et al., 2021	*Crtc1^–/–^* mice	New *Crtc1^–/–^* mouse model generated by the CRISPR/Cas9 system exhibiting an obese phenotype, but apparently independent of alterations in food intake or energy expenditure. Crucial role of CRTC1 in regulating lipid metabolism in adipose tissue during development.
Choong et al., 2013	Human, psychiatric patients and general population	First study showing an association of *CRTC1* polymorphisms with body mass index (BMI) and fat mass in humans.
Quteineh et al., 2016	Human, general population samples	*CRTC1* polymorphisms seem to play a role with obesity markers in individuals diagnosed with lifetime MDD rather than non-depressive individuals. No direct association of *CRTC1* polymorphisms with MDD in the three samples tested.
Lu et al., 2016	Human, meta-analysis in a large sample	*CRTC1* locus found in the 12 loci reaching genome-wide significance in the genome-wide association meta-analysis of body fat percentage in more than 100,000 individuals.
Rohde et al., 2019	Human, general population samples	DNA methylation levels of a CpG within the CRTC1 rs7256986 polymorphism and in a neighboring CpG were allele/genotype-dependent, suggesting a methylation quantitative trait locus (meQTL) in whole blood and adipose tissue. The presence of the SNP and/or DNA methylation correlated with CRTC1 gene expression, which in turn, related to BMI and fat distribution.
Delacretaz et al., 2019	Human, psychiatric patients with psychotropic treatment	Significant methylation changes observed in three *CRTC1* CpG sites in the blood of patients with early and important weight gain. One of these 3 CpG sites was significantly associated with early weight gain in patients carrying the G allele of rs4808844A > G, a SNP associated with this methylation site.
Wang et al., 2021	Human, 4 obese patients and 4 controls	Genome-wide DNA methylation analysis and pyrosequencing confirmation revealed that the methylation levels of 2 CpG sites in *CRTC1* were significantly changed in patients with obesity compared with normal controls.

Several human studies evaluated the association of *CRTC1* single nucleotide polymorphisms (SNPs) with obesity markers in the general population. To our knowledge, this association was first studied by Choong et al. (2013), who showed no significant association of the *CRTC1* SNP rs6510997 (a proxy of the rs3746266 G allele) with BMI, weight or waist circumference in the general population. Similarly, *CRTC1* locus did not reach genome-wide significance for BMI in the study of Locke et al. (2015), despite the very large sample size. Conversely, *CRTC1* SNP rs757318 was found, for the first time, to be associated with Body Fat percentage (BF%) and BMI in a genome-wide association study (GWAS) aiming at establishing a link between adiposity and cardiometabolic disease risk (Lu et al., 2016). This study reported that *CRTC1* SNP rs757318 had a more pronounced effect on BF% than on BMI, suggesting that BMI is an heterogeneous and less precise marker for adiposity, as it depends on both lean and fat mass. Interestingly, *CRTC1* locus showed a significant sex-specific interaction with a two to threefold larger effect in women than in men. However, the BF%-increasing *CRTC1* rs757318 allele was not associated with any of the cardiometabolic traits analyzed in this study. An interaction between *CRTC1* SNP and sex was also observed in a human study that examined whether the *CRTC1* polymorphism was associated with obesity markers in subjects with lifetime depression. Here, the *CRTC1* SNP rs6510997 was found to be negatively associated with BMI in women with a lifetime diagnosis of depression (Quteineh et al., 2016). Taken together these observations suggest that: (1) the association of CRTC1 locus with obesity markers is stronger when reliable markers of adiposity (such as BF%) are used, (2) it may exist a sex-specific effect, even though, the use of different *CRTC1* SNPs and different sample populations (general population versus psychiatric cohorts) complicate the interpretation of these GWAS studies. Moreover, due to the lack of knowledge about the biological effect of the tested SNPs on CRTC1’s function in humans (enhancement or decrease of CRTC1 activity in the brain), it is difficult to know whether the sex difference in energy balance regulation observed in *Crtc1^–/–^* mice is a specific feature of rodents.

Besides these human studies, the greatest contribution to the understanding of the regulatory role of CRTC1 in energy balance comes from animal experiments. A first study described the effects of the suppression of CRTC1 in the central control of food intake and energy expenditure (Altarejos et al., 2008). *Crtc1^–/–^* mice exhibited hyperphagia and body weight gain from early adult age with consequent development of obesity. The obese state of these mutant mice was also accompanied by reduced locomotor activity, lower energy expenditure and impaired sensitivity to leptin and insulin. However, it should be emphasized that there are gender differences regarding the vulnerability to develop obesity in this mouse model. Indeed, we showed that *Crtc1^–/–^* females are not hyperphagic and are less prone to gain weight as compared to mutant males, whose hyperphagia appears to be related to altered circadian locomotor activity (Rossetti et al., 2017). Moreover, CRTC1 seems necessary to protect against hepatic steatosis, which is strongly associated with obesity and metabolic syndrome, and to modulate leptin’s glucoregulatory actions in insulin-dependent diabetes (Kim et al., 2015; Kim, 2016). Whether CRTC1 is solely involved in the central control of energy balance or whether it also plays a role in peripheral organs is still unclear. A new *Crtc1^–/–^* mouse model generated by the CRISPR/Cas9 system also exhibits an obese phenotype, but it appears to be independent of alterations in food intake or energy expenditure (Hu et al., 2021). In this complete knockout mouse model, CRTC1 seems to be rather implicated in the regulation of lipid metabolism in adipose tissue during development. The creation of *Crtc1* conditional knockout (cKO) mouse models should greatly help to decipher the specific role of CRTC1 in various brain regions and cell types. To date, there has been only one article reporting the use of a Crtc1 cKO mouse model (Matsumura et al., 2021). This study showed that mice with a ventromedial hypothalamus-specific knockdown of *Crtc1* are sensitive to high-fat diet-induced obesity, exhibiting hyperphagia and increased body weight gain, but have a normal feeding behavior with a control chow diet.

Energy balance regulation depends on the interaction of many brain pathways that finely adapt food intake and energy expenditure to maintain energy homeostasis. After feeding, the release of leptin from adipocytes promotes satiety by acting on arcuate neurons in the hypothalamus (Myers et al., 2009; Pan and Myers, 2018). The activity of leptin depends on the stimulation of hypothalamic neurons releasing anorexigenic peptides and on the concomitant inhibition of neurons that secrete orexigenic peptides (Schwartz et al., 2000; Williams and Elmquist, 2012; Yeo and Heisler, 2012). The current working hypothesis suggests that CRTC1 contributes to leptin anorexigenic activity by modulating the expression of genes that participate in energy homeostasis. Accordingly, experimental evidence shows that leptin is able to activate the CRTC1/CREB pathway through different manners. This adipokine promotes CREB phosphorylation through the activation of the JAK2/STAT3 intracellular cascade (Catalano et al., 2009). In addition, leptin enhances CRTC1 nuclear translocation (Altarejos et al., 2008), possibly through the inhibition of AMP-activated kinase (Minokoshi et al., 2004) and increased activity of anorexigenic POMC neurons (Cowley et al., 2001). On the other hand, disrupted leptin signaling in leptin–deficient *ob/ob* mice is associated with increased amounts of phosphorylated, inactive CRTC1 in the cytoplasm of hypothalamic neurons (Altarejos et al., 2008).

Among the CRTC1/CREB-regulated genes, *Cart* (cocaine-amphetamine related transcript) is of particular importance for the suppression of food consumption, as it mediates leptin activity in the hypothalamus. In line with these molecular interactions, obese leptin-deficient *ob/ob* mice have a lower expression of *Cart* in the hypothalamus, and peripheral leptin injection normalizes hypothalamic CART peptide levels in these obese mice (Duan et al., 2007). Moreover, the capacity of CRTC1 to mediate the stimulating effect of leptin on *Cart* expression is supported by the increased plasmatic levels of leptin, reduced leptin receptor (*LepRb*) mRNA expression and lower *Cart* transcription in the arcuate nucleus of obese *Crtc1^–/–^* mice. Conversely, younger hyperphagic but not obese *Crtc1^–/–^* mice that are still leptin-sensitive, show comparable number of phospho-STAT3 positive cells in the arcuate nucleus after leptin infusion and similar leptin receptor (*LepRb*) and *Cart* mRNA levels as compared to lean wild-type mice (Altarejos et al., 2008; Rossetti et al., 2017). These data validate the functional interplay between leptin and CRTC1/CREB pathway, but point out the fact that the impaired *Cart* expression is probably just a consequence of leptin resistance and obesity, and that the lack of CRTC1 has likely broader effects on energy balance regulation by affecting other relevant genes.

In this regard, BDNF is an interesting candidate. Although first implicated in the etiology of MDD, this member of the neurotrophin family of growth factors was later recognized as a key component of mechanisms regulating energy intake and expenditure (Rios, 2013; Marosi and Mattson, 2014; Xu and Xie, 2016). According to a physiological role of BDNF in energy control, the expression of *Bdnf* and its receptor *TrkB* is sensitive to the nutritional state. Indeed, food deprivation reduces BDNF mRNA expression in the rat ventromedial hypothalamus, whereas glucose injection rapidly induces *Bdnf* and *TrkB* transcription in the same region (Xu et al., 2003; Unger et al., 2007). Complementary studies showed that the central injections of BDNF in rodents (i.c.v. or directly in the ventromedial or paraventricular hypothalamus) lead to reduced food intake, increased energy expenditure and body weight loss (Toriya et al., 2010). On the contrary, different genetic manipulations in mice, resulting in lower expression of either *Bdnf* or its *TrkB* receptor, cause hyperphagia and induce signs of metabolic syndrome including leptin and insulin resistance, dyslipidemia and hyperglycemia (Kernie et al., 2000; Rios et al., 2001). Finally, BDNF is also playing a role in the hedonic regulation of food intake involving the mesolimbic dopamine system. Consumption of palatable, high-fat food (HFF) influences *Bdnf* and *TrkB* expression in the dopaminergic neurons of the ventral tegmental area (VTA), whereas BDNF depletion in the VTA affects the reward function of the mesolimbic dopamine system and leads to excessive intake of palatable HFF, but not of standard chow, and to increased body weight under HFF conditions (Cordeira et al., 2010). This study suggests that an alteration of BDNF signaling may interfere with the activity of the mesolimbic dopamine pathway, leading to reward deficiency and compensatory overeating of palatable food.

In parallel to this preclinical research, human genetic studies also support the involvement of BDNF in energy balance regulation. The functional loss of one copy of the BDNF gene reduces serum BDNF concentration, increase *ad libitum* food intake and provokes severe early-onset obesity (Gray et al., 2006). Moreover, a missense mutation in the *NTRK2* gene (human TrkB receptor gene) that prevents the regular activity of the receptor was identified in patients exhibiting overweight and severe obesity (Yeo et al., 2004). Finally, a functional polymorphism of the *BDNF* gene (BDNF Val66Met), which impedes the correct secretion and signaling of BDNF, was correlated with obesity predisposition in children and adolescents (Beckers et al., 2008; Skledar et al., 2012).

Consistent with the importance of the CRTC1/CREB pathway for *Bdnf* transcription, *Crtc1^–/–^* mice have lower mRNA levels of both *Bdnf* and *TrkB* in different brain regions as compared to wild-type mice. Precisely, the analysis of the multiple *Bdnf* splice variants in the hypothalamus of these mice revealed a significant reduction in the *Bdnf* mRNA for the exon I and IV (Breuillaud et al., 2012). A recent study using mice with selective knockdown of BDNF production from either promoter I, II, IV, or VI showed that disruption of BDNF from promoter I or II, but not IV or VI, induces hyperphagic obesity (McAllan et al., 2018). The energy balance dysregulation observed in *Crtc1^–/–^* mice might thus be related to a decrease of *Bdnf* exon I mRNA in the hypothalamus. Collectively, these findings suggest that BDNF could represent one of the most important CRTC1/CREB downstream genes that contribute to the energy homeostasis. However, a direct causality between *Crtc1* deficiency and reduced hypothalamic *Bdnf* transcripts in the development of obesity is still elusive, and therefore, further molecular and behavioral researches are required.

Although less studied than *Bdnf*, another potential interesting CRTC1/CREB target gene is the neuron-derived orphan nuclear receptor 1 (*Nor-1*, also known as *Nr4a3*). This gene controls food intake in the arcuate nucleus by integrating leptin and glucocorticoid signaling (Kim et al., 2013). Interestingly, its expression in the arcuate nucleus is downregulated both in young and old *Crtc1^–/–^* mice (Rossetti et al., 2017).

Over the last decade, a compelling number of studies showed that chronodisruption (or circadian rhythm alteration) in humans severely increases the risk for developing both psychiatric and metabolic diseases (Barandas et al., 2015; Albrecht, 2017; Logan and McClung, 2019; Xie et al., 2019). Accordingly, the deletion of genes belonging to the mouse circadian clock machinery, notably *Bmal1* and *Clock*, leads to hyperphagia, metabolic alterations and obesity (Rudic et al., 2004; Turek et al., 2005; Albrecht, 2012). Many environmental and genetic factors contribute to the maintenance of the circadian rhythm and allow the optimization of multiple biological functions along the daily night and day cycle (Dietrich and Horvath, 2013; Challet, 2019). It is long time known that photic inputs activate the CREB pathway in neurons of the suprachiasmatic nucleus (SCN) of the hypothalamus. This effect is likely due to variations in Ca^2+^ and cAMP levels in the SCN neurons induced by increased glutamate release from the fibers of the retino-hypothalamic tract that connects neural cells in the retina to the SCN cells (Ginty et al., 1993; Obrietan et al., 1999). This hypothesis is in accordance with the fact that both cAMP and Ca^2+^ levels oscillate within the SCN (Prosser and Gillette, 1991; Ikeda et al., 2003). In line with the role of CREB in the light-evoked circadian clock entrainment in SCN neurons, CREB-phosphorylation oscillates along the day in the mouse SCN and is higher during the first part of the light cycle (Travnickova-Bendova et al., 2002). More recently, it was shown that the dephosphorylation of CRTC1 and its subsequent nuclear accumulation peaks during the light part of the daily cycle (Table 3). This effect was specific for CRTC1, because CRTC2 did not show any variation (Sakamoto et al., 2013). Besides, the interaction of the CRTC1/CREB pathway with the circadian system is bidirectional, because complementary evidence exists about the control of some core clock genes by this transcriptional pathway. The suppression of CREB functionality in a tetracycline-inducible CREB repressor mouse strain leads to a significant reduction of both the expression of the circadian clock proteins PERIOD1 and PERIOD2 and the clock output hormones AVP and VIP in the SCN (Lee et al., 2010). Moreover, CRTC1 is involved in the negative feedback of the clock-resetting process that follows a light phase shift (jet lag) (Jagannath et al., 2013). In the SCN, light-activated CRTC1 increases SIK1 that phosphorylates CRTC1 and inactivates it. With this feedback mechanism, CRTC1 limits the transcription of *Period1* that promotes clock resetting. Thereby, CRTC1 may slow down clock resetting and prevent an abrupt desynchrony between the master clock located in the SCN and the other peripheral clocks. This buffering system could protect the SCN clock (master clock) from inappropriate phase shifting caused not only by exposure to sudden changes in light intensity during the night, but also by abnormal Zeitgeber stimuli (such as food intake, physical activity, and temperature changes) that are all known to affect its resetting. Because the SCN master clock controls the other clocks located in other brain regions and in peripheral organs, an abrupt shift of the SCN clock would severely compromise normal physiological functions of the organism [see discussion in Jagannath et al. (2013)]. More recently, Jagannath et al. (2021) also highlighted the role of the CREB/CRTC1 pathway in adenosine-mediated integration of both light and sleep signaling to regulate circadian timing in mice. Another study in *Drosophila* showed that CRTC mutation affects the circadian oscillation of clock genes expression producing a phase delay especially in PERIOD and TIMLESS proteins. This work highlights that the interaction between CRTC homologs and the circadian clock machinery has likely an ancestral origin and selectively evolved among different species (Kim et al., 2016).

**TABLE 3:** Summary of studies implicating CRTC1 in circadian rhythms regulation.

References	System	Findings
Sakamoto et al., 2013	C57BL/6 mice	Rhythmic expression of CRTC1 in the suprachiasmatic nucleus (SCN). CRTC1 expression was detected in the middle of the subjective day, with limited expression during early night, and late night expression levels intermediate between mid-day and early night levels. During early and late subjective night, a brief light pulse induced strong nuclear accumulation of CRTC1 in the SCN. Evidence of CRTC1-mediated *Per1* gene regulation.
Jagannath et al., 2013	C57BL/6 mice	In the SCN, light-activated CRTC1 increases SIK1 levels, which in turn phosphorylate and inactivate CRTC1. This feedback mechanism limits the transcription of *Per1* that promotes clock resetting. CRTC1 slows down clock-resetting and prevents an abrupt desynchrony between the master clock located in the SCN and the other peripheral clocks.
Kim et al., 2016	*Drosophila*	Light-independent role of *Drosophila* CRTC in sustaining circadian behaviors. Crtc null mutation dampens light-independent oscillations of TIMELESS (and not PERIOD) in the clock neurons.
Rossetti et al., 2017	*Crtc1^–/–^* mice	*Crtc1^–/–^* males have a hyperphagic behavior that is restricted to the diurnal (resting) phase of the light cycle during which they have a higher locomotor activity possibly due to circadian rhythms alteration.
Jagannath et al., 2021	C57BL/6 mice	Adenosine, encoding sleep history, acts upon the circadian clockwork via adenosine A1/A2A receptor signaling through the activation of the Ca^2+^-ERK-AP-1 and CREB/CRTC1-CRE pathways to regulate the clock genes Per1 and Per2. These signaling pathways converge upon and inhibit the same pathways activated by light. Circadian entrainment by light is thus systematically modulated on a daily basis by sleep history.

**FIGURE 2:**
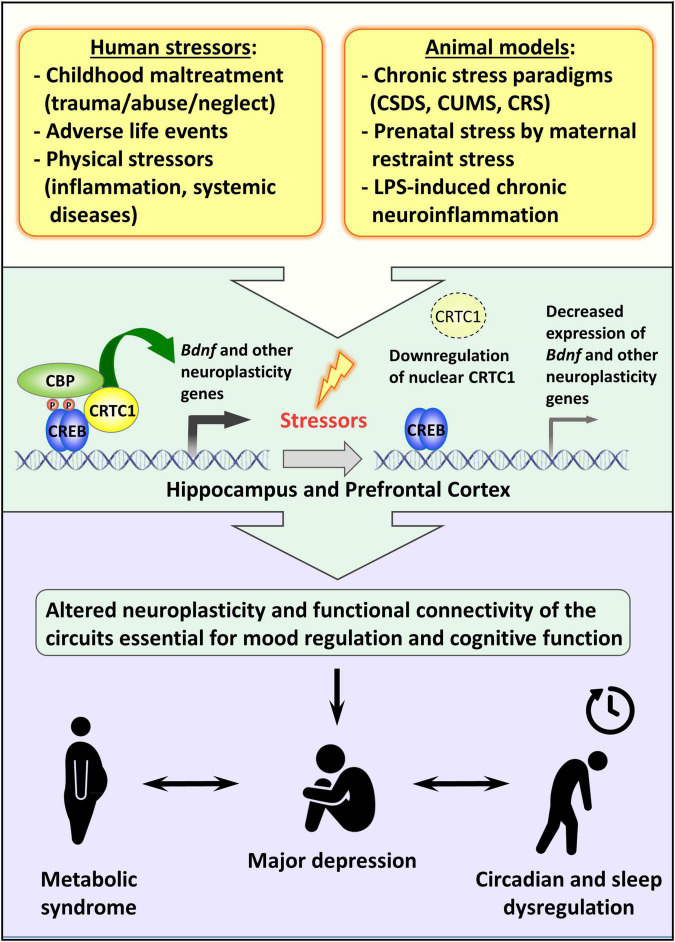
Possible role of CRTC1 in the pathogenesis of depression and associated disorders. Preclinical animal models of depression suggest that life stressors decrease CRTC1 levels in the prefrontal cortex and hippocampus, which triggers an altered neuroplasticity and functional connectivity related to the pathogenesis of depression and associated metabolic syndrome and chronodisruption.

Although the effective participation of CRTC1 in the entrainment of circadian clock *in vivo* and the consequent effect on the circadian energy homeostasis are not completely understood, the behavioral observation of *Crtc1^–/–^* mice suggested that the obesity of mutant males is due to overeating during the resting phase of the light cycle and to circadian alteration of spontaneous locomotor activity (Rossetti et al., 2017). Remarkably, the depressive-like phenotype of *Crtc1^–/–^* mice and their concomitant altered circadian rhythms and feeding behavior strongly suggest that CRTC1 is an important player in the central regulation of mood, circadian rhythms, and energy balance – all of which are dysregulated in MDD (see Figure 2).

## Conclusion and Perspectives

This review highlighted the increasing evidence of the pivotal role of the transcription coactivator CRTC1 in MDD and comorbid obesity. Mice lacking CRTC1 exhibit depressive-like endophenotypes (Breuillaud et al., 2012; Meylan et al., 2016a,b) and an altered regulation of feeding behavior resulting in hyperphagic obesity only in males (Rossetti et al., 2017). Interestingly, this gender-specific alteration of energy balance is associated with a dysregulated circadian locomotor activity. More research is needed to understand why *Crtc1^–/–^* female mice are less affected and whether the altered circadian activity of the males correlates, for instance, with a reduced average duration of sleep and modified sleep patterns. Possibly related to these metabolic and circadian alterations, MRS studies with *Crtc1^–/–^* male mice suggest that CRTC1 plays a critical role in regulating brain neuroenergetics. A possible limitation to the face validity of the *Crtc1* knockout mouse model of depression should, however, be considered, as the constitutive lack of CRTC1 may have neurodevelopmental effects that may influence the adulthood phenotypes. Future studies with *Crtc1* conditional knockout mouse models that may be combined with viral approaches should address this issue and assess a putative role of CRTC1 during neurodevelopment. Human *CRTC1* polymorphisms were associated with obesity markers (BMI, fat mass and distribution, eating behavior) in psychiatric cohorts and in the general population (Choong et al., 2013; Lu et al., 2016; Quteineh et al., 2016; Rohde et al., 2019). However, these polymorphisms were not directly associated with depression and further investigations are required to find a possible link between CRTC1 and MDD in humans.

Recent reports highlighted the instrumental role of ventral hippocampal CRTC1 in mediating chronic stress- and LPS-induced depressive-like behaviors, and antidepressant response in mice (Jiang B. et al., 2019; Ni et al., 2019). Jiang B. et al. (2019) compellingly shed light on the specific increased hippocampal expression of SIK2 triggered by chronic stress in two different paradigms (CUMS and CSDS). The higher levels of SIK2 increase CRTC1 phosphorylation and its retention in the cytoplasm, thus leading to a less active CRTC1-CREB-BDNF pathway and depressive-like symptoms. In the LPS-induced sickness behavior model of depression, Ni et al. (2019), uncovered the important regulatory role of hippocampal CRTC1 in controlling neuroinflammation and the CRTC1-CREB-BDNF-VGF pathway. Future research should characterize the molecular mechanisms of SIK2 induction in various chronic stress paradigms. The effects of CRTC1, BDNF, and VGF downregulation on hippocampal neuroplasticity should also be better defined and correlated with depressive-like behaviors. We and others showed that CRTC1 is required for conventional antidepressants response, but it is still unclear whether CRTC1 is involved in the sustained long-term effects of the novel rapid-acting antidepressants targeting glutamate neurotransmission. Finally, it remains to be determined whether the same CRTC1-regulated genes are jointly involved in MDD-related neuroplasticity processes, brain energy metabolism, circadian rhythms, and central control of energy balance, or whether different subsets of CRTC1 target genes are independently implicated. The transcription factor CREB is considered as a primary hub for activity-driven neuronal gene expression (Benito et al., 2011). Likewise, as a coactivator of CREB and possibly other transcription factors, CRTC1 could be a primary hub for a network of genes involved in the central regulation of mood, circadian rhythms, and energy balance. Future studies aiming at deciphering the underlying mechanisms through which downregulated CRTC1 functions commonly impinge on mood, circadian rhythms and energy balance regulation should lead to better insight into the etiopathogenesis of MDD and comorbid obesity, and may provide new therapeutic targets.

## Author Contributions

CR, AC, LG, and J-RC drafted and edited the manuscript. All authors read and approved the manuscript.

## Conflict of Interest

The authors declare that the research was conducted in the absence of any commercial or financial relationships that could be construed as a potential conflict of interest.

## Publisher’s Note

All claims expressed in this article are solely those of the authors and do not necessarily represent those of their affiliated organizations, or those of the publisher, the editors and the reviewers. Any product that may be evaluated in this article, or claim that may be made by its manufacturer, is not guaranteed or endorsed by the publisher.
